# The Effectiveness of Brain Injury Family Intervention in Improving the Psychological Well-Being of Caregivers of Patients With Traumatic Brain Injury: Protocol for a Randomized Controlled Trial

**DOI:** 10.2196/53692

**Published:** 2024-03-14

**Authors:** Siti Aminah Omar, Nor Sheereen Zulkefly, Firdaus Mukhtar

**Affiliations:** 1 Department of Psychiatry Faculty of Medicine Universiti Teknologi MARA Sungai Buloh Malaysia; 2 Department of Psychiatry Faculty of Medicine and Health Sciences Universiti Putra Malaysia Serdang Malaysia

**Keywords:** traumatic brain injury, TBI, caregiver, randomized controlled trial, psychological well-being, Malaysia

## Abstract

**Background:**

Globally, traumatic brain injury (TBI) is recognized as one of the most significant contributors to mortality and disability. Most of the patients who have experienced TBI will be discharged home and reunited with their families or primary caregivers. The degree of severity of their reliance on caregivers varies. Therefore, the task of delivering essential care to the patients becomes demanding for the caregivers. A significant proportion of caregivers expressed considerable burden, distress, and discontentment with their lives. Therefore, it is critical to comprehend the dynamic of TBI and caregivers to optimize patient care, rehabilitation, and administration. The effectiveness of the Brain Injury Family Intervention (BIFI) program tailored for caregivers of patients with TBI has been widely proven in Western countries. However, the impact is less clear among caregivers of patients with TBI in Malaysia.

**Objective:**

This study aims to assess the effectiveness of BIFI in reducing emotional distress and burden of care, fulfilling the needs, and increasing the life satisfaction of caregivers of patients with TBI at government hospitals in Malaysia.

**Methods:**

This is a 2-arm, single-blinded, randomized controlled trial. It will be conducted at Hospital Rehabilitasi Cheras and Hospital Sungai Buloh. In total, 100 caregivers of patients with TBI attending the neurorehabilitation unit will be randomized equally to the intervention and control groups. The intervention group will undergo the BIFI program, whereas the control group will receive standard treatment. Caregivers aged ≥18 years, caring for patients who have completed >3 months after the injury, are eligible to participate. The BIFI program will be scheduled for 5 sessions as recommended by the developer of the module. Each session will take approximately 90 to 120 minutes. The participants are required to attend all 5 sessions. A total of 5 weeks is needed for each group to complete the program. Self-reported questionnaires (ie, Beck Depression Inventory, Positive and Negative Affect Schedule, Caregiver Strain Index, Satisfaction With Life Scale, and Family Needs Questionnaire) will be collected at baseline, immediately after the intervention program, at 3-month follow-up, and at 6-month follow-up. The primary end point is the caregivers’ emotional distress.

**Results:**

The participant recruitment process began in January 2019 and was completed in December 2020. In total, 100 participants were enrolled in this study, of whom 70 (70%) caregivers are women and 30 (30%) are men. We are currently at the final stage of data analysis. The results of this study are expected to be published in 2024. Ethics approval has been obtained.

**Conclusions:**

It is expected that the psychological well-being of the intervention group will be better compared with that of the control group after the intervention at 3-month follow-up and at 6-month follow-up.

**Trial Registration:**

Iranian Registry of Clinical Trials IRCT20180809040746N1; https://irct.behdasht.gov.ir/trial/33286

**International Registered Report Identifier (IRRID):**

RR1-10.2196/53692

## Introduction

### Background and Rationale

Traumatic brain injury (TBI) is defined as any injury sustained by the head as a result of blunt or penetrating trauma, acceleration or deceleration forces [1]. It remains as a leading cause of mortality and morbidity worldwide [2]. In Malaysia, despite investment in various preventive efforts, the incidence of TBI continues to increase yearly [3]. In 2009, as many as 166,768 trauma cases were recorded in 8 hospitals in Malaysia, most cases (76.8%) being road traffic accidents [4]. A recent study reported an extremely high cost of treatment for patients with TBI in Malaysia. The estimated annual cost of treatment for 49 patients with TBI was as high as MYR 1.5 million (US $313840) [5]. In the long term, this would lead to an adverse impact on the social and economic development of the country.

The effects of TBI depend largely on the severity and location of the injury and the age and personality of the patient [1]. TBI will affect their self-care ability, employment capacity, and social functioning. Upon discharge from hospitals, most patients with moderate to severe levels of TBI would need to move back in with their families to receive the care they need [6,7]. Patients with severe TBI are unlikely to return to their previous employment as they require a significant amount of care [8]. As most of the patients with TBI are highly dependent, their caregivers are tasked with providing the necessary physical care for them. The caregivers are also constantly involved in any ongoing rehabilitation of the patients, for example, encouraging them to perform physiotherapy exercises and reminding them to take medication. Moreover, the caregivers need to deal with difficult behaviors and challenging emotional states of patients with TBI [9-12]. These extra demands can be unfavorable to the health and well-being of the caregivers.

Many studies have highlighted the importance of understanding the dynamics of TBI on caregivers. There is an extensive body of research on the effect of TBI on caregivers globally [13-22]. However, local data are scarce, as only a few studies have been conducted to assess the effects of TBI on Malaysian caregivers [23-25]. The available studies have found that most TBI caregivers in Malaysia reported high burden and poor life satisfaction as a result of caregiving activities [25]. Similarly, there is also a lack of information regarding the needs of caregivers of patients with TBI in developing countries. It was suggested that caregivers of patients with TBI should be provided a postdischarge rehabilitation program to reduce their burden as caregivers [25]. A systematic review has suggested that caregivers of patients with TBI should be prioritized in TBI rehabilitation [26], as evidence abounds on the benefits of intervention programs tailored for caregivers toward patients with TBI [27-30].

In Malaysia, there is a lack of specific or structured intervention programs focusing on the psychological well-being of caregivers of patients with TBI. Identifying caregivers at an increased risk of burden is important for preventing emotional distress and caregiver burden to improve care for both patients and caregivers. Therefore, it is important to design a proper intervention plan to improve the quality of life of caregivers of patients with TBI. Ultimately, this will result in better care and management of patients with TBI.

### Conceptual Framework

This study is based on 2 major theories, namely, the Family System Theory (FST) and Cognitive Behavior Therapy (CBT) [31].

The first theory, FST, assumes that the whole family is interconnected to one another [32]. For instance, if a family member is affected by TBI, the whole family system will also be affected [31]. Patients with TBI would most likely depend on their family members for activities of daily living, routine follow-up, rehabilitation, and financial support. This would increase the burden on the caregivers. Family members would also need to look for coping resources to overcome their problems [13,33,34]. As a result of the sudden changes in the family’s functioning, family members often reported a lack of coping skills, in addition to high levels of burden, anxiety, and emotional distress [17,33,35-40], thus resulting in a decrease in psychological well-being [19,41-43]. Therefore, Brain Injury Family Intervention (BIFI) incorporates family therapy techniques such as normalization and validation to assist patients with TBI and their families.

The second theory, CBT, is widely known for its use in treating various types of psychological disorders [44]. Several studies have applied CBT to provide psychological interventions to patients with TBI and their caregivers [28,45-50]. A systematic review revealed that CBT has a significant impact on improving the psychological well-being of TBI caregivers [32,45]. CBT would equip caregivers of patients with TBI with strategies to deal with psychological problems such as depression and anxiety [44]. A specific component of CBT would also be implemented in BIFI that covers important aspects such as psychoeducation, problem-solving skills, management of emotions, setting of realistic goals and expectations, and stress management.

### Aims of This Study

This study aims to assess the effectiveness of BIFI in reducing emotional distress and burden of care, besides fulfilling the needs and increasing the life satisfaction of caregivers of patients with TBI in selected hospitals.

The hypotheses of this study are as follows:

1. There is a significant association between the sociodemographic and clinical characteristics of patients with TBI and the emotional distress, burden of care, needs, and life satisfaction of caregivers of patients with TBI.
2. There is a significant difference in the mean scores of caregivers’ emotional distress, burden of care, needs, and life satisfaction between the intervention and control groups before the intervention, after the intervention, at 3-month follow-up, and at 6-month follow-up.
3. The sociodemographic and clinical characteristics of the patients are predictors of the intervention outcomes.

## Methods

### Study Design

This is a 2-arm, single-blinded, randomized controlled trial (RCT). All participants will be randomly assigned to the intervention group or control group. Only the investigators will be aware of the treatment allocation.

### Study Setting

**Figure 1:**
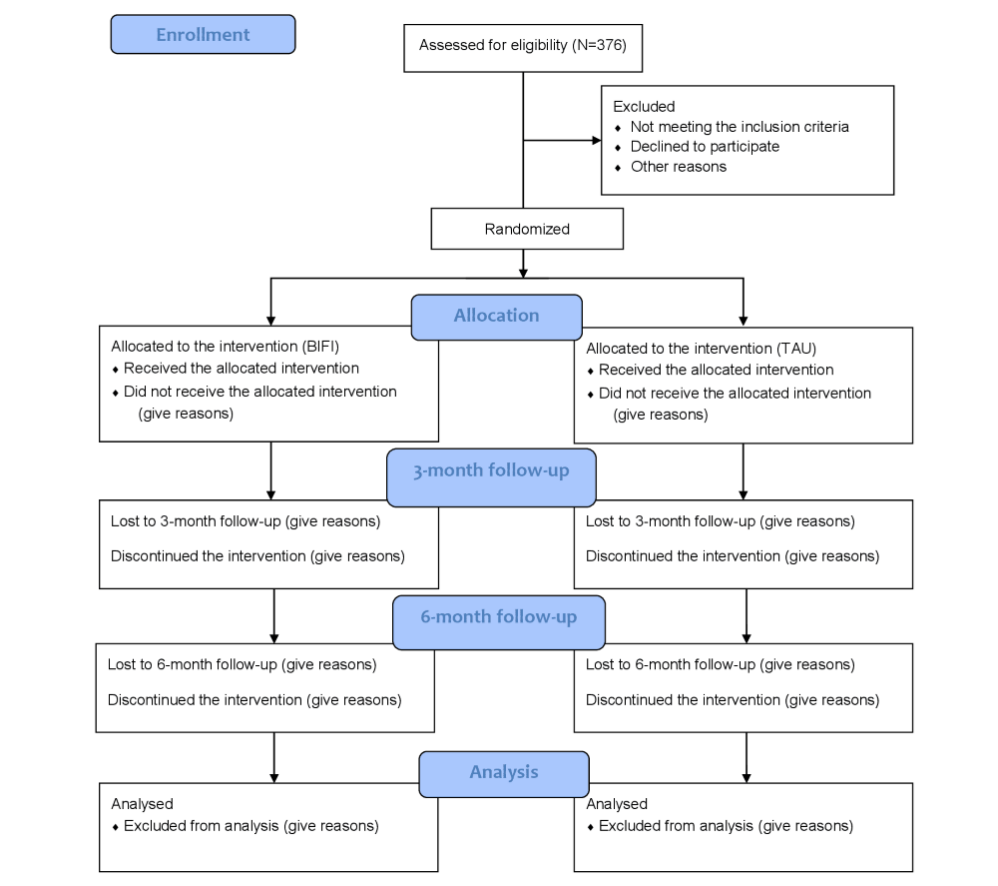
Research flowchart. BIFI: Brain Injury Family Intervention; TAU: treatment as usual.

This study will be conducted at Hospital Rehabilitasi Cheras (HRC) and Hospital Sungai Buloh (HSB). These hospitals are the main referral hospitals for patients with TBI. A specific room in each site will be used for data collection. Figure 1 outlines the study flow according to CONSORT (Consolidated Standards of Reporting Trials) 2010.

### Participants

A total of 100 participants were recruited for this study. Of the 100 participants, 50 (50%) were randomly assigned to the intervention and control groups, respectively. All caregivers of patients with TBI who attend follow-up with patients with TBI at the Neuro Rehabilitation outpatient clinic of HRC and HSB were screened for eligibility to participate in this study.

### Inclusion and Exclusion Criteria

To be eligible, caregivers of patients with TBI must be citizens or permanent residents of Malaysia and be aged ≥18 years, regardless of their race, ethnicity, and sex. The participants must be able to read or write in Bahasa Malaysia or English. The caregivers can be the parents, spouses, siblings, sons or daughters, or relatives of the patients with TBI. Only 1 caregiver per patient with TBI can be recruited for the program. Time since injury for the patient with TBI must be >3 months and time spent on caring for the patients must be at least 2 hours per day. All types of injury severity were included. However, paid caregivers were excluded.

### Withdrawal Criteria

The participants may choose to withdraw at any point of time without any penalty. The participants may be withdrawn if the investigator believes that it is harmful or risky for the participants to continue.

### Sample Size Estimation

Sample size estimation is based on the formula for clinical superiority trial [51]:

N=2x (Z_1–α_ + Z_1–β_)^2^ × S^2^ / (δ–δ_0_).

The sample size is calculated with 80% power (Z_1–β_=0.845) at a significance level (α) of .05, with 95% CI (Z_1–α_=1.96), 25% dropout rate, and medium size effect of 0.60. The parameters used in the formula will be based on a previous study [45], including δ^2^=9.355 (pooled SD) and depression (Beck Depression Inventory [BDI] score) δ–δ_0_=5.86 (mean difference). According to the calculation, a total of 50 participants per arm will be needed for this study.

The parameter used to compute the prevalence of caregivers of patients with TBI was taken from the paper titled “Life satisfaction and strain among informal caregivers of patients with traumatic brain injury in Malaysia” [25].

### Sample Size Calculation for Prevalence Studies

In this formula, n=sample size, z=z statistic for the level of confidence, P=expected prevalence, and d^2^=allowable error. This formula assumes that P and d^2^ are decimal values but would also hold true if they are percentages, except that the term, 1–P, in the numerator would become 100–P [52]:

N=Z^2^
*P* (1–P) / d^2^

Percentage of caregiver’s burden=57.4%, level of significance=5%, power=80%, and d=0.05:

N=(1.96)^2^ 0.574 (1 – 0.574) / 0.05^2^

N=376

This study aimed to assess eligibility for at least 376 caregivers of patients with TBI.

### Allocation

#### Allocation Sequence Generation

Methods of random allocation are used to ensure that all study participants have the same chance of allocation to the treatment or control group. Caregivers of patients with TBI who are eligible and consent to participate will be number coded before randomization. A simple random sampling method using computer-generated random sampling will be used to assign participants to the intervention group or control group. The procedure would be performed by following the 1:1 allocation format. Randomization will be performed by the principal investigator. All participants will have equal chance of being assigned to the intervention or control group.

#### Contamination Bias

To minimize contamination bias, the intervention program will be conducted outside their follow-up clinic time. The intervention program will be conducted at the seminar room of the designated hospitals. Participants are strongly encouraged not to disclose the program materials or discuss them with other caregivers of patients with TBI outside the program.

#### Blinding

The method of blinding in RCT is used to ensure that there are no differences in the way in which each group is assessed or managed and therefore minimize bias. In this study, only the principal investigator is aware of the treatment allocated to the participants.

### Statistical Analysis

Data analysis will be performed using SPSS software (version 24.0; IBM Corp). Descriptive statistics will be used to describe the characteristics of participants. Continuous data will be reported as means and SDs or as medians and IQRs. Categorical data will be reported as percentages and frequencies. The comparison of the means between the 2 groups will be performed using a paired samples 2-tailed *t* test. In addition, 1-way ANOVA and repeated measure ANOVA will be conducted to identify any significant differences in mean scores of emotional distress (BDI), caregiver’s needs (Family Needs Questionnaire [FNQ]), burden of care (Caregiver Strain Index [CSI]), and life satisfaction (Satisfaction With Life Scale [SWLS]) before the intervention, after the intervention, at 3-month follow-up, and at 6-month follow-up between the intervention and control groups. Multivariate regression analysis will be used to determine the predictors of intervention outcomes.

The intention-to-treat analysis will be performed accordingly. Assumptions of normality and homogeneity of variance will be conducted and adjusted where necessary.

### Program Assessment and Translation

The BIFI manual has undergone several stages of thorough forward and backward translation, content review, and revision by a panel of local experts. The expert panel included 2 clinical psychologists, 1 certified translator (psychology content translator), and 2 rehabilitation physicians. All the panels had experience with working in the related fields for >5 years.

On the basis of the experts’ feedback and comments, a few changes were made to the module. The first change was the use of a more simple Malay language. The second change was related to the pictures used in the material. The experts suggested changing to universal pictures, so that they can be adapted locally.

The final product of the manual was revised to match the local population and to ensure the intervention’s fidelity.

### Pilot Study

A pilot study was conducted to assess the feasibility of the BIFI program among caregivers of patients with TBI in Malaysia. In total, 10 caregivers of patients with TBI participated, and only 8 (80%) managed to complete all 5 sessions. A caregiver withdrew owing to work commitments and another withdrew owing to personal issues. Challenges included (1) punctuality of the participants, (2) duration of the sessions, and (3) homework material.

Overall, participants were satisfied with the content and delivery of the program. Some suggested to increase the duration of the sessions and to reduce the amount of homework given. All feedback was taken into consideration, and slight alterations were performed to accommodate the participants and the program.

### Intervention

BIFI will be the main intervention tool. It is a structured intervention module specifically for patients with TBI and their caregivers, developed by Professor Dr Jeffry Kreutzer and colleagues from Virginia Commonwealth University, United States. This module is based on CBT [44] and FST [53].

This module consists of several objectives: (1) providing patients and caregivers with fundamental information about brain injury, (2) helping caregivers to better understand the effects of brain injury, (3) teaching patients and caregivers about problem-solving skills, (4) teaching coping strategies, (5) recognizing progress and personal strengths and helping them to access community and professional resources, and (6) teaching effective communication skills.

BIFI was designed to be implemented in 5 sessions, with 90 to 120 minutes for each session. In total, 2 or 3 topics will be covered in each session. Using a standardized and family-focused intervention, BIFI was found to be beneficial for caregivers of patients with TBI both immediately and at 3 months after the intervention [31,54,55]. Furthermore, another study also showed that patients with TBI and caregivers reported high ratings of helpfulness, goal attainment, and satisfaction regarding the BIFI program [55].

Several measures will be taken to ensure that all participants comply with the intervention program. The program would also be conducted during weekends to accommodate the schedule of caregivers of patients with TBI. They are also allowed to choose any day during weekends at their convenience. However, they are required to complete all 5 sessions within the stipulated time frame.

### Control Group

The control group or treatment-as-usual group will not receive any additional treatment during the study period. Participants in the control group will receive the usual treatment at their respective hospitals. According to the Malaysian Clinical Practice Guidelines for early management of head injury, all patients who have been discharged are recommended to attend follow-up sessions at the hospital [56]. It is recommended that patients with moderate and severe head injuries are scheduled for routine clinic follow-up. However, patients with a mild head injury can be followed up via clinic visits or telephone calls. Apart from the routine follow-up, other programs tailored for patients with TBI and their caregivers are also considered as treatment as usual in this RCT. For example, the Acquired Brain Injury Rehabilitation Unit in HRC would offer a program known as “Return to Work” for suitable patients and caregivers. A similar program is also available in HSB.

### Outcomes

#### Overview

The outcomes of this study will be assessed using self-report questionnaires at four periods: (1) baseline, (2) 5 weeks (after the treatment), (3) 3-month follow-up, and (4) 6-month follow-up. The schedule of enrollment, interventions, and assessments are presented in Table 1.

**Table 1:** Schedule of enrollment, interventions, and assessments.

				Study period										
				Enrollment		Allocation		After allocation				Follow-up		
Time point				–t_1_		0		Baseline t_1_		5-week intervention t_2_		3 months t_3_		6 months t_4_
**Enrollment**														
		Eligibility screen	✓											
		Informed consent	✓											
		Randomization	✓											
		Allocation			✓									
**Interventions**														
	BIFI^a^ and TAU^b^													
	TAU only													
**Assessments**														
	Sociodemographic data				✓									
	BDI^c^						✓		✓		✓		✓	
	PANAS^d^						✓		✓		✓		✓	
	CSI^e^						✓		✓		✓		✓	
	FNQ^f^						✓		✓		✓		✓	

^a^BIFI: Brain Injury Family Intervention.

^b^TAU: treatment as usual.

^c^BDI: Beck Depression Inventory.

^d^PANAS: Positive and Negative Affect Schedule.

^e^CSI: Caregiver Strain Index.

^f^FNQ: Family Needs Questionnaire.

#### Primary Outcomes

The primary outcome for this study is the TBI caregiver’s emotional distress, which will be measured using two scales: (1) BDI and (2) Positive and Negative Affect Schedule (PANAS). The measures are described in the Methods section.

#### Secondary Outcomes

The secondary outcomes will include (1) burden (CSI), (2) life satisfaction (SWLS), and (3) caregiver’s needs (FNQ). The measures are described in the Methods section.

### Data Collection and Time Frame

Recruitment for potential participants was conducted by the rehabilitation physicians at both hospitals, who are also the coinvestigators of the study. They screened for potential participants among the caregivers of patients with TBI who attend regular rehabilitation therapy and follow-up at their clinics. These caregivers were then invited to participate in this study. If the caregiver was interested in learning more about the study, they were led to another room, where the investigator explained the study in great detail and answered any questions that the caregivers had. At this stage, a patient information sheet about the nature of the study was provided to the potential participants.

The investigator then left the room for 10 to 15 minutes to allow the caregiver to read the information and to think about whether they would like to participate in the study. If they expressed interest in participating, they were asked to sign the consent form before completing the questionnaire. The same questionnaire used at baseline will be distributed immediately after the intervention, at 3-month follow-up, and at 6-month follow-up. The questionnaire will take approximately 30 to 40 minutes to complete. Data collection was performed between December 2018 and December 2020.

### Measures

#### BDI Questionnaire

The original version of BDI is a self-reporting questionnaire consisting of 21 items on a 4-point scale. It is assessed using a Likert scale ranging from 0 (symptom not present) to 3 (symptom very intense). The total score can range from 0 to 63. BDI is used to measure the main symptoms of depression such as mood, pessimism, sense of failure, self-dissatisfaction, guilt, punishment, self-dislike, self-accusation, suicidal ideas, crying, irritability, social withdrawal, indecisiveness, body image change, work difficulty, insomnia, fatigability, loss of appetite, weight loss, somatic preoccupation, and loss of libido [57]. Respondents who scored between 0 and 9 are considered negative for depression. In contrast, those who score >9 are screened positive for depression, whereby a score between 10 and 18 indicates mild to moderate depression, score between 19 and 29 indicates moderate to severe depression, and score between 30 and 63 indicates severe depression (60). The BDI test is widely used globally, and its content, concurrent, and construct validity have been tested. High concurrent validity ratings are detected between BDI and other depression instruments such as the Minnesota Multiphasic Personality Inventory and the Hamilton Depression Scale. Correlation rating of 0.77 was obtained between the inventory and psychiatric ratings. BDI also showed high construct validity with the medical symptoms it measures. The study by Beck and Steer [58] reported a coefficient α rating of 0.92 for patients at outpatient clinics and 0.93 for college students. BDI has been translated into the Malay language and validated for use among the Malaysian population. Internal consistency (Cronbach α) ranged from 0.71 to 0.91, and the validity of BDI-Malay was deemed as satisfactory [59].

#### PANAS Questionnaire

PANAS comprises 2 mood scales that measure the positive affect (PA) and negative affect (NA), respectively. PANAS is used to assess the relationship between positive and negative effects on personality traits. Each of the PA and NA scales consists of 10 items that define their meanings. Respondents need to answer 20 items on a 5-point scale that ranges from 1 (very slightly or not at all) to 5 (extremely). The total score generated will range between 10 and 50, with low scores indicating low (positive or negative) affect and high scores indicating high (positive or negative) affect. The reliability and validity of PANAS were moderately good [60]. For the PA scale, the Cronbach α coefficient was between 0.86 and 0.90, and for the NA scale, it was between 0.84 and 0.87. Over 8 weeks, the test-retest correlations were between 0.47 and 0.68 for PA and between 0.39 and 0.71 for NA. PANAS also had strong validity with other measures of general distress and dysfunction, depression, and anxiety [61]. The Malay-translated version of PANAS had Cronbach α coefficient of 0.73 [62].

#### CSI Questionnaire

CSI is a self-rated, 13-item questionnaire that measures strain related to care provision. It consists of 5 major domains related to employment, financial, social, time, and physical aspects. The items can be answered as yes (score=1) or no (score=0). The maximum score for the questionnaire is 13. A score >7 is categorized as “having strain,” whereas a score <7 is defined as “no strain.” There is no age limit for the individuals who could be assessed with the tool**.** CSI has been translated into the Malay language, and the Cronbach α coefficient for the 13-item CSI-Malay was 0.79, indicating good internal consistency reliability of the scale [63].

#### SWLS Questionnaire

SWLS evaluates the respondents’ agreement with 5 statements on overall satisfaction with life (eg, in most ways, my life is close to my ideal). It uses a 7-point Likert scale ranging from 1 (strongly disagree) to 5 (strongly agree), giving rise to a range of scores between 5 and 35. A score of 20 is considered the neutral point of the scale. Scores between 5 and 9 indicate that the respondent is extremely dissatisfied with life. In contrast, scores between 31 and 35 show that the respondent is extremely satisfied. SWLS is reported as having excellent internal consistency (Cronbach α=0.88) and good test-retest reliability (*r*=0.68). SWLS has been translated into the Malay language and validated among the Malaysian population [64,65]. The Malay SWLS has been found to have good internal consistency (Cronbach α=0.83).

#### FNQ Questionnaire

FNQ is a self-administered questionnaire consisting of 37 items. It was designed to determine the family needs of patients with TBI. The family members will rate the importance of each need on a scale ranging from 1 (not important) to 4 (very important). FNQ is divided into 6 areas, namely, health information, emotional support, instrumental support, professional support, community support network, and involvement with care. It has been proven to have good content and construct validity and good internal consistency Spearman-Brown split-half reliability at 0.75 [66].

### Study Procedure

All the screening procedures to identify the participants based on inclusion and exclusion criteria were performed before the intervention program by rehabilitation physicians in the respective hospitals. The recruitment started in November 2018 and was continued until the required sample size was achieved. The BIFI program will be conducted in the Malay or English language depending on the needs of the caregivers. The participants in the intervention group will be divided into smaller groups of 10 people each. Each group will receive a scheduled time and date to attend the program at the hospital. The program will be conducted by the principal investigator, who is also a clinical psychologist. The clinical psychologist must at least have 5 years of experience.

At the baseline of the program (T1), all participants are required to complete the questionnaires (BDI, CSI, PANAS, FNQ, and SWLS). The same questionnaire will be distributed immediately after the intervention program (T2), at 3-month follow-up (T3), and at 6-month follow-up (T4). The average time needed to complete the questionnaire is 30 to 40 minutes. The follow-up sessions (T3 and T4) will be scheduled by the coinvestigators. The session will be conducted at the respective hospitals by the coinvestigators to prevent any bias during follow-up. The participants were also encouraged not to share the intervention materials with other caregivers until data collection is completed.

The intervention group will undergo the BIFI program. The BIFI program will be scheduled for 5 sessions as recommended by the developer of the module. Each session will take approximately 90 to 120 minutes. The participants are required to attend all 5 sessions. A total of 5 weeks is needed for each group to complete the program, and 2 groups will be scheduled every week. All the sessions will start with an overview of the topic and end with the summary and homework assignments. Participants will be required to complete the homework given according to the module. This homework will then be reviewed by the principal investigator in the subsequent session.

For the control group that receives the usual standard treatment, the caregivers will need to complete the questionnaires at similar time points as the intervention group.

### Patient Involvement

Patients were involved during the early stage of cultural adaptation of the intervention program. The patients were invited to give their comments and feedback and review all the materials. Their valuable feedback was taken into account to ensure this program is suitable to the current culture and population.

### Ethical Considerations

#### Approval

This study has been reviewed by the Medical Research and Ethics Committee Malaysia, Ministry of Health Malaysia (NMRR 18-2253-42951). Ethics approval has been obtained from the National Medical Research Register (NMRR-18-2253-42951-IIR) for the study to be conducted at the Ministry of Health settings. Please refer to Multimedia Appendices 1 and 2 for further details of Research Ethics comments and review. The approval letter was received on December 19, 2018. The recruitment period started in December 2018 and was completed on December 18, 2020.

#### Compensation

All the participants in the intervention group will be given incentives for attending the intervention program and completing the questionnaires. All participants are compensated with a travel token at the baseline (MYR 25), immediately after the program (MYR 25), at 3-month follow-up (MYR 25), and at 6-month follow-up (MYR 25).

#### Consent

The investigator will explain the potential risks and benefits of involvement to the participants using an information leaflet before they determine whether to participate in the study. They might ask the researcher any questions they may have regarding the study before deciding whether to participate. Once the investigator is confident that they have understood the potential risks and benefits of participating, the participant will be asked to sign a consent form. The consent forms are obtained in the written format during primary data collection and secondary data analysis is allowed to proceed without additional informed consent.

#### Data Management

Consent forms and paper copies of the questionnaire will be stored separately in a locked filing cabinet at University Teknologi MARA. After data collection is complete, they will be transferred to a locked filing cabinet. They will be maintained by the principal investigator for 10 years according to university regulations. Data will be accessible to the researchers and anyone authorized by Universiti Teknologi MARA to conduct a research audit.

Electronic copies of the data will be maintained by the principal investigator. These electronic files will not contain any personal identifying information and will not contain the identifying code that links the paper copies of the questionnaires with the consent forms. They will be stored only on password-protected electronic devices. Once the thesis submission and other publications are completed, these files will be destroyed. The files will be accessible to the principal investigator, the research team, and anyone authorized by Universiti Teknologi MARA to conduct a research audit.

No personal identifying details, such as names and contact details, will be recorded on the questionnaire; they will appear only on the consent form. The questionnaires will be linked to the consent forms by a unique code appearing on both documents. No digital record of the personal identifying details will be maintained, and these details will not be included in the data file. The report of the findings will also not include any such details.

### Dissemination Plan

The findings of this study will be published in an academic or medical journal, and they will be presented at academic conferences. Only the research team has access to the data. As with any anonymously obtained data, the participants will not be named in any of the study’s reports or publications. Permission from the Medical Research and Ethics Commission will be sought before publication.

## Results

The participant recruitment process began in January 2019 and was completed in December 2020. A total of 100 participants were enrolled in this study, of whom 70 (70%) caregivers are women and 30 (30%) are men. We are currently at the final stage of data analysis. The result of this study is expected to be published in 2024.

## Discussion

### Anticipated Findings

In this study, we will be evaluating the effectiveness of BIFI in reducing the emotional distress and burden of care, fulfilling the needs, and increasing the life satisfaction of caregivers of patients with TBI. A total of 100 caregivers were recruited in this study. Most of the caregivers are women (70/100, 70%) and the remaining are men (30/100, 30%). The age range of the caregivers was between 22 and 55 years, with a mean age of 39.85 (SD 8.184) years. Most caregivers were Malay (65/100, 65%), followed by Chinese (21/100, 21%), Indians (12/100, 12%), and other races (2/100, 2%). Initial analysis showed promising result where there was significant reduction in emotional distress among caregivers (intervention group) immediately after the program and at the 3-month follow-up as compared with the control group.

### Limitations

This study also has limitations. It was observed that during the implementation of the program, most participants (90/100, 90%) requested the program to be conducted during the weekends, whereas others wanted it to be conducted during the weekdays. This was because some of them needed to arrange for the patient’s care if they were to attend the program. Hence, the program was conducted during weekends and weekdays to accommodate their request. The participants were allowed to choose when they wanted to attend the program. Owing to this issue, the intervention program took a long period to complete.

It is also important to address that data collection was conducted during the COVID-19 pandemic. The intervention program was halted for several months owing to Movement Control Order by the Malaysian Government. Therefore, participants were hesitant to visit the research site owing to fear of contracting COVID-19; the social distancing policy exacerbated this difficulty. It is hoped that future studies might to consider implementing intervention programs over the web to ease the participants. Web-based intervention program is another emerging approach and is more suited to current trends. However, web-based intervention versus physical intervention is still debated, and more studies are needed to answer this question.

### Conclusions

To the best of our knowledge, this study would be among the first to use RCT methods to assess the effectiveness of BIFI in improving the psychological functioning of caregivers of patients with TBI in Malaysia. Perhaps, this module could be incorporated into the *Return to Work* program as standard clinical care and be made available to all. It is hoped that the results will provide more knowledge and scientific evidence to improve the rehabilitation services for patients with TBI and their caregivers.

## Appendix

Multimedia Appendix 1 Research ethics comments.

Multimedia Appendix 2 Peer-review report by the Medical Research and Ethics Committee Malaysia.

## Abbreviations

BDI: Beck Depression Inventory
BIFI: Brain Injury Family Intervention
CBT: Cognitive Behavior Therapy
CSI: Caregiver Strain Index
CONSORT: Consolidated Standards of Reporting Trials
FNQ: Family Needs Questionnaire
FST: Family System Theory
HRC: Hospital Rehabilitasi Cheras
HSB: Hospital Sungai Buloh
NA: negative affect
PA: positive affect
PANAS: Positive and Negative Affect Schedule
RCT: randomized controlled trial
SWLS: Satisfaction With Life Scale
TBI: traumatic brain injury

## Data Availability

The data of this study are available upon request from the corresponding author, and it is subject to approval from the Ministry of Health Malaysia and Universiti Teknologi MARA.
