# Supplementary material for: The Effectiveness of Brain Injury Family Intervention in Improving the Psychological Well-Being of Caregivers of Patients With Traumatic Brain Injury: Protocol for a Randomized Controlled Trial
Source: JMIR Res Protoc. 2024 Mar 14;13:e53692. doi: 10.2196/53692 (PMC10979341; doi:10.2196/53692)
Supplement: Multimedia Appendix 1 [file resprot_v13i1e53692_app1.pdf]

JAWATANKUASA ETIKA & PENYELIDIKAN PERUBATAN
(*Medical Research & Ethics Committee*)
KEMENTERIAN KESIHATAN MALAYSIA
d/a Institut Pengurusan Kesihatan
Jalan Rumah Sakit, Bangsar
59000 Kuala Lumpur

Tel.: 03-2287 4032/2282 0491/2282 9085
03-2282 9082/2282 1402/2282 1449
Faks: 03-2282 0015

Ruj.Kami:(13)KKM/NIHSEC/ P18-1980
Tarikh: 28-November-2018

Siti Aminah Binti Omar
University Teknologi Mara (UITM) - Sungai Buloh Campus

Tuan/Puan,

NMRR-18-2253-42951 (IIR)
A randomized controlled trial on the effectiveness of Brain Injury Family Intervention (BIFI) in improving psychological well-being of Traumatic Brain Injury caregivers at Sungai Buloh Hospital and Cheras Rehabilitation Centre.

Dengan hormatnya dimaklumkan bahawa Jawatankuasa Etika & Penyelidikan Perubatan, Kementerian Kesihatan Malaysia, di mesyuaratnya pada 27-November-2018 telah menilai permohonan Tuan/Puan dan telah memberikan ulasan berikut :

The MREC Committee has reviewed the study and the following explanations and corrections / revisions are required prior to the final decision:

1.0 Protocol

1.1 Insert the protocol identifying number and date
1.2 Insert the name and address of the sponsor.
1.3 Insert the investigator/s name and institution in full
1.4 Insert information/ reviews on the known risks and potential benefits of the program
1.5 Please provide details whether the risk acceptable for the expected benefits of the programs
1.6 Insert the study objective(s) clearly
1.7 Insert information on what the ethical issues in the study are and how are the issues addressed
1.8 Clarify the study design including all procedures appropriate
- As the study objective is to study the burden of care and yet to participate in the study, the carer in the intervention group has to come to the hospital for the training course at their own expenses while at the same time have to look for another person to take care of patients at home, will this increases their burden and stress?? Please clarify and justify the objective clearly

1.9 Insert explanation on the measures taken to minimize bias such as randomization, blinding, maintenance of randomization codes, and procedures for breaking codes / State how is randomization conducted, who maintains randomization codes, and procedures for breaking code
- What type of program the control group will be received
- Explain how is it possible to blind the investigator
- Explain how would randomisation limits the possible contaminations of

the intervention and control group

1.10 Insert the criteria for suspending or terminating the study

1.11 Clarify the calculation of sample size as in methodology, it stated the participant are expected to drop out but in the sample calculation there is no dropout rate considered

1.12 Provide information on the subject inclusion and exclusion criteria

- Clarify if the mental stability of the care giver is being addressed

1.13 Insert explanation on the process, place and timing for obtaining informed consent

1.14 Insert the subject withdrawal criteria apart of self-withdrawal

1.15 State who is providing insurance or payment for treatment of study related injuries

- Clarify the insurance taken to cover the subject throughout the study (it will not be covered by the crown indemnity as subject is not a patient of the hospital)

1.16 Insert statement on the publication policy for protecting the confidentiality of subject's personal information

2.0 Patient Information Sheet (PIS)/ Informed Consent Form

2.1 Insert the name of the sponsor

2.2 Insert the purpose of the study clearly

- Explain what is TBI caregivers

2.3 It is noted that study will be a double blind- doctor /researcher& subject, However in protocol it stated as a single blind. Please clarify and revise.

2.4 Explain what it means by "need to perform a screening test". Please state the test

2.5 Insert the subject's responsibilities clearly

- Please rephrase the section as the program stated in the study is referred as "product". (to avoid confusion) . Kindly get rid of information that not related to the program (do not copy exactly like the template given)

2.6 Insert information on the reasonably foreseeable risks or inconveniences of the study

2.7 Insert information if there is any compensation and/or treatment in the event of study-related injury/ies

- Clarify who is the sponsor/ what kind of insurance the subject will be covered (Para 13 stated about the sponsor coverage on any bodily injury or illness resulting from the study)

2.8 Insert the circumstances (if any) where the subject's participation may be terminated

2.9 State how is confidentiality of data is secured

- Clarify why is the data is being transmitted outside of the country and to whom it will be transmitted. If so, how the data is being transmitted

3.0 Others

3.1 NIL

Please indicate or highlight the changes made as per MREC comments in the revised document/s and complete the section on "Investigator's Response" in the REPORT ON FOLLOW-UP REVIEW OF STUDY DOCUMENTS appended with this letter.

2. Sehubungan dengan itu, semua dokumen yang lengkap dan telah diperbetulkan/baru hendaklah dimuatnaikkan melalui www.nmrr.gov.my tidak lewat daripada 20 hari bekerja (satu bulan kalendar) daripada tarikh surat ini. Sekiranya Tuan/Puan memerlukan sedikit lagi masa, sila maklumkan kepada pihak kami melalui e-mail ke mrecsec@nih.gov.my dengan segera sebelum berakhirnya 20 hari bekerja tersebut. Sekiranya tiada sebarang maklumbalas diterima sehingga 20 hari bekerja dari tarikh surat ini, maka permohonan penyelidikan ini dianggap telah terbatal dan tidak akan diproses seterusnya. Surat pembatalan penyelidikan akan dikeluarkan selepas itu.

Sekian, terima kasih.

BERKHIDMAT UNTUK NEGARA

Saya yang menjalankan amanah,

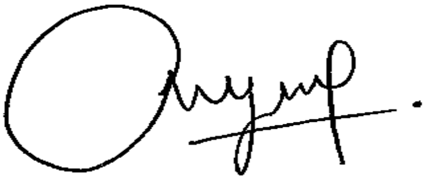

(DR ASYRAF SYAHMI BIN MOHD NOOR)

b.p Setiausaha
Jawatankuasa Etika & Penyelidikan Perubatan
Kementerian Kesihatan Malaysia
