# Supplementary material for: The Effectiveness of Brain Injury Family Intervention in Improving the Psychological Well-Being of Caregivers of Patients With Traumatic Brain Injury: Protocol for a Randomized Controlled Trial
Source: JMIR Res Protoc. 2024 Mar 14;13:e53692. doi: 10.2196/53692 (PMC10979341; doi:10.2196/53692)
Supplement: Multimedia Appendix 2 [file resprot_v13i1e53692_app2.docx]

**APPENDIX II**

**REVIEW FORM FOR RESEARCH PROTOCOLS INVOLVING HUMAN SUBJECTS WITH MINIMAL RISK**

| **NMRR ID:** | NMRR-18-2253-42951 |
| --- | --- |
| **Study Protocol Title:** | A randomized controlled trial on the effectiveness of Brain Injury Family  Intervention (BIFI) in improving psychological well-being of Traumatic Brain  Injury (TBI) caregivers at two government hospital |
| **Principal Investigator:** | Siti Aminah Omar |
| **Date Protocol Received by Reviewer:** | 05/09/2018 |

| NOTE: | Please evaluate whether the ITEMS outlined below (**Section 1**) have been appropriately addressed in the study protocol. State any explanation or additional information required in the COMMENTS column. The middle column in the Table below MUST BE COMPLETED FOR ALL SECTIONS. Finalize your review by stating any additional comments in the OTHER COMMENTS. This section is to be completed for review and re-review. |
| --- | --- |

**SECTION 1. PROTOCOL REVIEW**

|  | ITEMS | State (Y) for ‘Yes’, (N) for ‘No’, (NR) if not relevant, (NS) if not sure, (NC) if not complete | COMMENTS |
| --- | --- | --- | --- |
|  | 1. **GENERAL INFORMATION** |  |  |
|  | - 1. Is study title appropriate? | Y |  |
|  | - 1. Is there a protocol identifying number and date? | N | To include protocol identifying number and date |
|  | - 1. Is the name and institution of investigator/s stated? | Y |  |
|  | 1. **BACKGROUND/LITERATURE REVIEW** |  |  |
|  | - 1. Is the literature review complete with sufficient information on the disease or medical condition studied, health issues addressed, the intervention/drug/device, previous findings of similar studies, methodologies used by similar studies, etc. | NC | To add literature review/information on:   - The TAU – to give examples/program run by the study sites - The BIFI program – why & how it was design (adopted or self-developed?), rationale behind the number of sessions or topic chosen, etc - The study subject’s selection criteria (>3 months care) – the effects on the duration of care for TBI patients on the psychological wellbeing of the caregivers as this may affect the effectiveness of the BIFI program |
|  | - 1. Is there an acceptable review of the potential benefits, of the study? | Y |  |
|  | 1. **OBJECTIVES AND PURPOSE** |  |  |
|  | - 1. Is the objective(s) clear and acceptable? | Y |  |
|  | 1. **STUDY DESIGN** |  |  |
|  | - 1. Is the study endpoint(s) or outcome(s) clearly stated and acceptable? | Y |  |
|  | - 1. Is the study design, including all data collection procedures, appropriate and acceptable? | NS | To clarify on:   - TAU – what’s the content? - BIFI program – language to be conducted? Conducted by who? Group or individual sessions? Arrangement for sessions’ appointment? - Follow-up appointment scheduled & conducted by who & where? - Data collection/usage for those withdrawn (statement provided in the PIS but not in protocol) |
|  | - 1. Is there acceptable rationale, description, specifications and justification for (a) study procedures and (b) survey questionnaires / interview questions? | NC | Refer 4.2 |
|  | - 1. Is the expected duration of subject participation acceptable? | NS | To clarify on the expected duration of subject participation (number & duration of sessions to attend, duration to answer questionnaire, etc) |
|  | - 1. Is the sequence and duration of all study activities including follow-up, acceptable and necessary? | NS | To clarify on the sequence and duration of all study activities including follow-up, especially for the TAU group |
|  | - 1. Is there acceptable procedure for monitoring of compliance of subjects? | N | To add information on the procedure for monitoring of compliance of subjects |
|  | - 1. Is there appropriate collection, storage and use of bio specimens, and personal information? | Y |  |
|  | 1. **SELECTION OF SUBJECTS** |  |  |
|  | - 1. Is the study population appropriate and clearly described? | NS | To clarify on:   - The study subject’s selection criteria (>3 months care) – the effects on the duration of care for TBI patients on the psychological wellbeing of the caregivers as this may affect the effectiveness of the BIFI program - Only 1 caregiver per TBI patient will be recruited or can allow multiple caregiver per TBI patient (if got > 1 caregiver) |
|  | - 1. Is there acceptable number of subjects to be enrolled including reason and calculation for sample size? | Y |  |
|  | - 1. Is there acceptable inclusion and exclusion criteria? | NS | Refer 5.1 |
|  | - 1. Is an informed consent/assent process required? | Y |  |
|  | 1. **ASSESSMENT OF RESULTS** |  |  |
|  | - 1. Is there acceptable specification of efficacy parameters, methods and timing for assessment, recording and analysis? | Y |  |
|  | 1. **ASSESSMENT OF RISK** |  |  |
|  | - 1. Is there **physical** (e.g. temporary dizziness, pain associated with venipuncture, etc)**, psychological** (e.g. episodes of depression, confusion, or hallucination resulting from drugs, feelings of stress, guilt, loss of self-esteem, etc)**, social (e.g.** alcohol or drug abuse, mental illness, illegal activities, sexual behaviour, actual or potential delinquents, schizophrenics, etc)**, and economic (loss of earnings from participation**, loss of job etc) risk involved? (Suggest to recommend MREC to review if YES) | NC | To clarify/add:   - Psychological risk when answering questionnaire/attending sessions? - Extra time needed to participate in study when answering questionnaire/attending sessions? - Extra cost incurred to participate in study when answering questionnaire/attending sessions? |
|  | 1. **SURVEY TOOL** |  |  |
|  | - 1. Is the instrument appropriate? | Y |  |
|  | - 1. Has the instrument been appropriately translated and/or validated? | NC | To clarify the validity of BM version of PANAS |
|  | 1. **STATISTICS** |  |  |
|  | - 1. Is there an acceptable statistical plan and methods for data analysis? | Y |  |
|  | 1. **CONFIDENTIALITY AND SECURITY OF SOURCE DOCUMENTS AND STUDY DATA** |  |  |
|  | - 1. Is there acceptable means for protecting privacy and confidentiality of personal information? | Y |  |
|  | - - 1. Is there a statement that study data to be returned or informed to subject, if the research results are validated and potentially relevant to the clinical management of the subject? | N | To add statement whether study data will be returned or informed to subject |
|  | - 1. Is there acceptable duration and means of storage and archival of medical records and study data? | Y |  |
|  | - - 1. Is it stated that study data is destroyed after period of storage? | NC | To add statement whether paper copy of study data will be destroyed after period of storage |
|  | 1. **PUBLICATION POLICY** |  |  |
|  | - 1. Is there a suitable publication policy, if publication is planned for protecting the confidentiality of subjects’ personal information? | NS | To add a clear statement on the publication policy |
|  | 1. **INVOLVEMENT OF VULNERABLE SUBJECTS** |  |  |
|  | - 1. Are minors involved as subjects? | NR |  |
|  | - - 1. If minors are involved, is there appropriate assent and parental agreement form?   ***Consider MREC review if data collected or interaction is more than minimal risk.** | NR |  |
|  | - 1. Is there any involvement of other vulnerable subjects (e.g. pregnant women, prisoners, children, cognitively impaired persons, students and employees, minorities, economically and/or educationally disadvantaged, AIDS/HIV+ subjects, terminally ill subjects).   ***Consider MREC review if data collected or interaction is more than minimal risk.** | NR |  |
|  | - - 1. Is there appropriate protection for the vulnerable subjects? | NR |  |
|  | 1. **MISCELLANEOUS** |  |  |
|  | - 1. Is the grammar and language acceptable? | Y |  |

| **ADDITIONAL DOCUMENTS REQUIRED FROM INVESTIGATOR (if any):**   - **Study products – discrepancy on the availability of study product in PIS & protocol** |
| --- |
| **OTHER COMMENTS (if any):**   - **To countercheck both PIS & protocol before submission as there are discrepancy of information between both documents** |

**SECTION 2:** **RECOMMENDATIONS**

Note: Please tick [/] the appropriate decision outlined below and submit completed form into NMRR system.

**Decision**: (tick [/] what is appropriate)

| [ ] | To recommend MREC approve without revision |
| --- | --- |
| [ / ] | To recommend MREC approve after revision |
| [ ] | To recommend MREC review (if study is more than minimal risk) |
| [ ] | Withdraw (poor protocol, major revisions, etc) |

| **HRRC REVIEWER** |  | 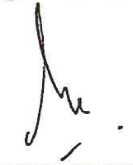Signature | *HRRC Review Board, Hospital Rehabilitasi Cheras, Kuala Lumpur* |
| --- | --- | --- | --- |
| Date: 16/09/2018 |  | Name |  |
| **HRRC SECRETARIAT** |  | Signature  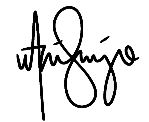 | *HRRC Secretariat, Hospital Rehabilitasi Cheras, Kuala Lumpur* |
| Date: 18/09/2018 |  | Name |  |
